# Adaptive locomotion during subtle environmental changes in younger and older adults

**DOI:** 10.1038/s41598-022-16436-4

**Published:** 2022-07-20

**Authors:** Ryota Sakurai, Kentaro Kodama, Yu Ozawa

**Affiliations:** 1 Research Team for Social Participation and Community Health, Tokyo Metropolitan Institute of Gerontology, 35-2 Sakae-cho, Itabashi-ku, Tokyo, 173-0015 Japan; 2 University Education Center, Tokyo Metropolitan University, 1-1 Minami-Osawa, Hachioji-shi, Tokyo, 192-0397 Japan; 3 Faculty of Sport Sciences, Waseda University, 2-579-15 Mikajima, Tokorozawa, Saitama, 359-1192 Japan

**Keywords:** Risk factors, Neurology

## Abstract

For older adults especially, to perform everyday activities safely, adaptive locomotion that adjusts basic locomotion pattern according to the environmental features is critical. It is unknown, however, whether their locomotor patterns can be modified when there are subtle environmental changes. We examined adaptive limb movements, focusing on obstacle avoidance and age-related changes during such situations. Younger (102, with a mean age of 27.5 years) and older (101, with a mean age of 78.3 years) participants walked across one obstacle (150 mm height) four different times. The obstacles were then covertly raised or lowered by 10% of the baseline obstacle height (i.e., 165 mm for ascending and 135 mm for descending conditions), and participants were asked to repeat the activity. We measured leading and trailing foot clearances, the vertical distances between toe tips and the upper edge of the obstacle. In the ascending condition, both groups adjusted and raised their limb clearance according to the obstacle height change. Alternatively, foot clearance of the leading limb for the lowered obstacle did not change among the older adults, whereas it changed in the young adults (lowered their clearance). No changes were observed in the trailing foot clearance for the descending conditions in either age group. Our results suggest that when facing environmental changes that compromise safe mobility, individuals can adapt leading limb movement based on subtle environmental changes, irrespective of age. In case of other changes (i.e., in low-risk situations), however, the ability of adaptive locomotion may be affected by aging.

## Introduction

Locomotor patterns are adjusted based on visual information about environmental properties at a distance to avoid future risk, which includes tripping on or colliding with obstacles and falling^1,2^. When facing a challenging situation, for example, an obstacle that blocks our travel path, we naturally stop walking, step over, or avoid it. Such locomotion, modifying basic movement patterns for propulsion in response to environmental constraints, such as uneven terrain, is known as adaptive locomotion^3^. Adaptive locomotion to adjust one’s movement and navigate a cluttered environment is critical in performing everyday activities safely and efficiently. This is especially true for older adults whose physical functioning declines with age, leading to increased risk of falling.

Many studies have investigated the adaptive locomotion involved in stepping over an obstacle, the most common adaptive locomotion, using several environmental conditions because stepping over is important to maintain a safe daily life^4–7^. Interestingly, some studies demonstrated that older adults adapted their movements excessively compared to young adults^8–10^. In a previous study, for instance, where the participants stepped over obstacles with three obviously different heights (10%, 20%, and 30% of the participants’ leg length), greater increased foot clearance with raised obstacle height was observed in the older candidates compared to the young adults^11^. This is not a surprising result as older adults likely stepped over higher obstacles more safely to avoid potential accidents. Results of a recent experiment requiring participants to step over a 26-cm high visible and stationary obstacle reported lower contact rates in middle-aged and older adults compared to young adults^12^, although obstacle contacts were more frequent in older adults compared to younger adults under time-constrained conditions in each of these studies^4^.

Although such adaptations have been confirmed among older adults when they face obvious environmental changes, it is unknown whether their locomotor patterns can be adjusted when they face more subtle ones. It has been suggested that highly automated or skilled behavior, including locomotion under familiar conditions without dual tasking, requires less awareness of environmental features, implying that such behaviors might be adapted without attention^13,14^. For example, pedestrians even adapt their locomotion to environmental features in more visually challenging environments, such as a crowded city, and successfully avoid an obstacle in their path, despite not being aware of the presence of the obstacle^15^. To the best of our knowledge, however, few reports have determined whether achievement of adaptive behavior to stationary obstructions, performed without awareness, occurs in the process of stepping over an obstacle.

Correct adaptation of subtle environmental changes, such as a series of steps of slightly different heights, including outdoor stairs or cord covers, can reduce fall hazards. On the contrary, if older adults cannot adapt to a subtle change, such as a raised obstacle, they may trip due to stepping over it as if it were a low obstacle before changing. Understanding the nature of the adaptive limb movement during obstacle avoidance in response to subtle environmental changes may help detect the underlying risk of falls among older adults. It may also help us understand how locomotion is adaptively controlled.

The purpose of this study was to clarify the nature of the adaptive limb movement while stepping over an obstacle, when faced with subtle environmental changes that were unnoticeable, and its age-related changes. Specifically, we examined whether younger and older adults adjusted their foot clearance when they stepped over covertly raised or lowered obstacles. Based on previous findings suggesting that older adults were likely to experience a decline in sensorimotor function encompassing all sensory, motor, and central integration and processing components involved with bodily movements^16^, we hypothesized that they would not be able to adjust their foot movement when the height of obstacles was subtly altered, while young adults would be able to do so. The findings of the present study can shed light on the interaction between environmental change and motor behavior.

## Results

During the ascending condition, two participants, one younger and one older (1.9% of the original participants, respectively) noticed the change in obstacle height during the test trial. Furthermore, one younger (in descending condition) and two older (in ascending condition) participants were excluded from the analysis because of unparseable data (e.g., missing due to technical issues). As a result, 102 young (51 and 51 for the ascending and descending conditions, respectively) and 101 older adults (49 and 52 for the ascending and descending conditions, respectively) were included in the final analysis.

Table 1 shows the participants’ characteristics stratified by the obstacle conditions. There were no significant differences in the characteristics of the participants between the ascending and descending conditions for both younger and older adults. Overall, the older participants in our study were free from instrumental activities of daily living (IADL) disability and had relatively good gait speed (mean gait speed in both groups > 1 m/s) and global cognitive function (mean Mini-Mental State Examination [MMSE] > 28) for their ages.

**Table 1:** Participant characteristics.

Variables	Younger adults			Older adults		
	Ascending condition (*n* = 51)	Descending condition (*n* = 51)	*p*-value	Ascending condition (*n* = 49)	Descending condition (*n* = 52)	*p*-value
Age, mean (SD)	26.8 (8.4)	28.2 (6.5)	0.340	78.3 (5.6)	78.3 (5.8)	0.988
Female, n (%)	25 (49.0)	30 (58.8)	0.321	42 (85.7)	41 (78.8)	0.368
LLL, mean, cm (SD)	80.6 (4.7)	81.3 (5.1)	0.471	75.1 (6.0)	76.8 (4.4)	0.104
GS, mean, m/s (SD)				1.37 (0.19)	1.34 (0.26)	0.616
MMSE, mean (SD)				28.4 (1.7)	28.6 (1.5)	0.372

*LLL* length of lower limb, *GS* gait speed, *MMSE* Mini-Mental State Examination.

### Ascending condition

**Figure 1:**
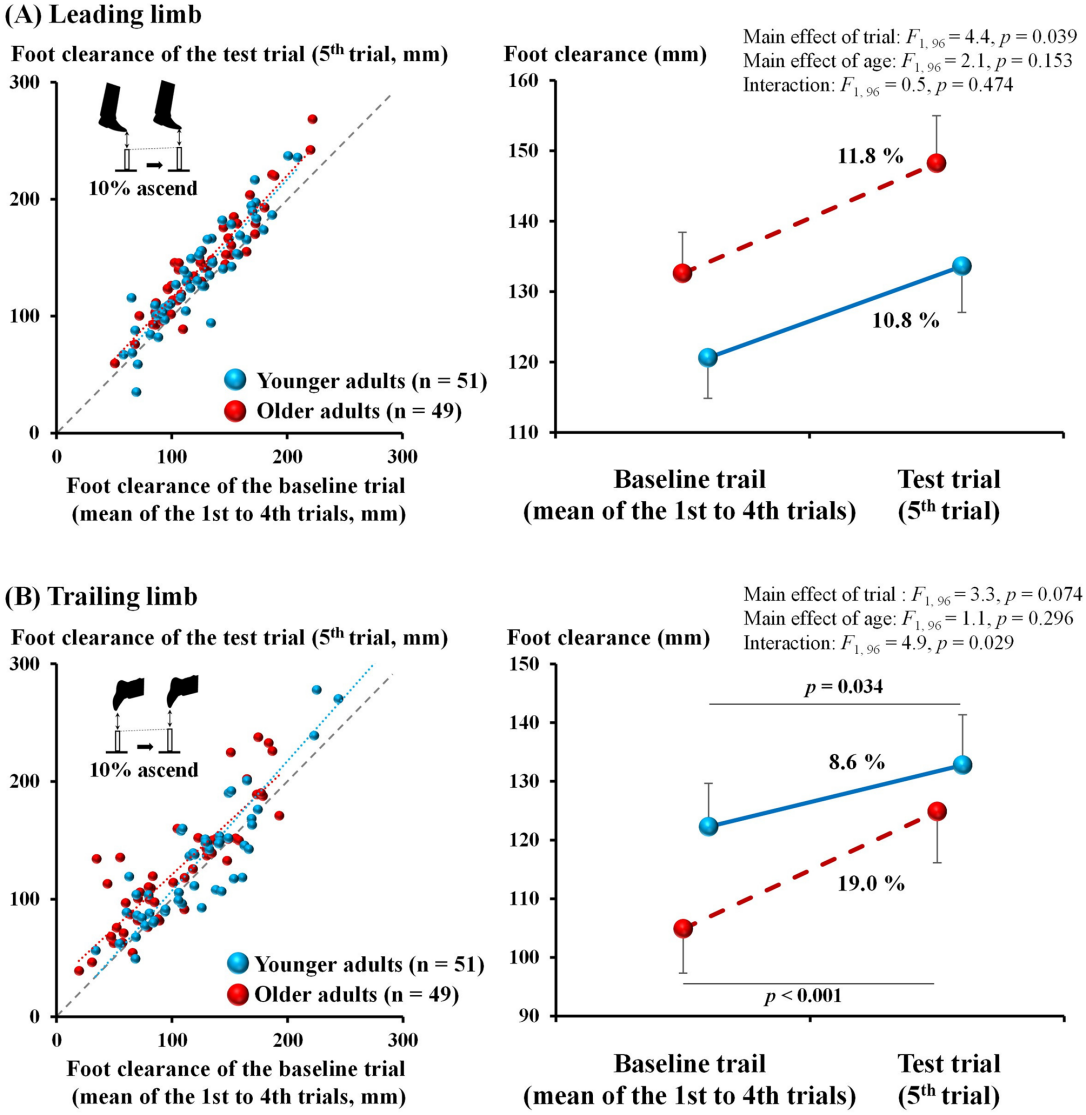
Comparison of the foot clearance in the ascending condition for the younger and older adults. Left side: scatter diagrams of the foot clearance of the baseline trial (mean of the 1st to 4th trials) and the test trial (5th trial) for the young and older adults. Data points above or below the diagonal line show participants who increased or decreased their foot clearance in the fifth trial. The light blue and red dashed lines indicate the regression lines of the young and older adults, respectively. Right side: line graphs of the foot clearance of the baseline trial (mean of the 1st to 4th trials) and the test trial (5th trial) for the young and older adults (error bars indicate standard errors). For both scatter and line diagrams, the upper scatter diagram (**A**) is for the leading limb and the lower scatter diagram (**B**) is for the trailing limb. Bars and error bars indicate adjusted mean actual values and standard errors.

Figure 1 shows the results of the clearance during the obstacle avoidance task in the ascending condition. Scatter diagrams of foot clearances of the baseline (e.g., mean foot clearance of first to fourth trials) and test (i.e., fifth trial) trials for the leading limb showed that most participants increased foot clearance in the test trial. Supporting these plots, the two-factor mixed analysis of covariance (ANCOVA) comparing foot clearance of the baseline and test trials with the age group factor revealed a significant main effect of the trail factor (*p* = 0.039, *η*^2^ = 0.044) but not the age factor (*p* = 0.153, *η*^2^ = 0.021), and no significant interaction between the two factors was observed (*p* = 0.474, *η*^2^ = 0.005), which indicated that both younger and older adults adjusted lead limb movement in response to changes in obstacle height. In accordance with the changing rate of the obstacle height (i.e., 10%), the mean changes in foot clearance in the test trial for younger and older adults were 10.8% and 11.8%, respectively.

For trailing limb (lower diagrams of Fig. 1), ANCOVA for log-transformed foot clearance showed no significant main effects (trial factor: *p* = 0.074, *η*^2^ = 0.033; age factor: *p* = 0.296, *η*^2^ = 0.011), although a significant interaction was observed (*p* = 0.029, *η*^2^ = 0.049). Post-hoc analyses showed significantly higher foot clearance for both younger (*p* = 0.034) and older adults (*p* < 0.001) in the test trial than in the baseline trial.

### Descending condition

**Figure 2:**
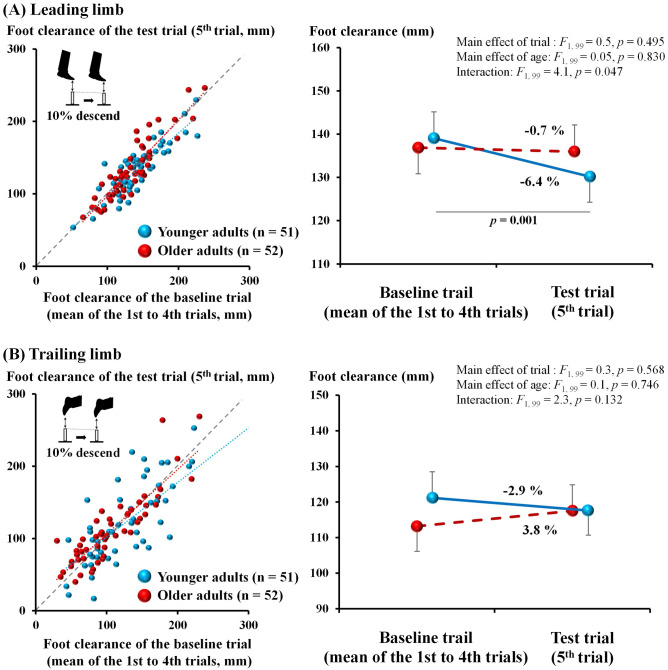
Comparison of the foot clearance in the descending condition for the younger and older adults. Left side: scatter diagrams of the foot clearance in the mean foot clearance of the first to fourth trials (i.e., baseline trial) and fifth trial (i.e., test trial) for the young and older adults. Data points above or below the diagonal line show participants who increased or decreased their foot clearance in the fifth trial. The light blue and red dashed lines indicate the regression lines of the young and older adults, respectively. Right side: line graphs of the foot clearance in the fifth trial and the mean foot clearance of the first to fourth trials for the young and older adults (error bars indicate standard errors). For both scatter and line diagrams, the upper scatter diagram (**A**) is for the leading limb and the lower scatter diagram (**B**) is for the trailing limb. Bars and error bars indicate adjusted mean values and standard errors.

Figure 2 shows results of the clearance during the obstacle avoidance task in the descending condition. Scatter plots depict the relationships between foot clearances in the baseline and test trials, displaying the result that younger rather than older adults decreased their foot clearance in the test trial compared to that in the baseline trial. The ANCOVA revealed a significant interaction (*p* = 0.047, *η*^2^ = 0.039) without any significant main effects (trial factor: *p* = 0.495, *η*^2^ = 0.005; age factor: *p* = 0.830, *η*^2^ = 0.001). In the post-hoc analyses, significantly lower foot clearance in the test trial than in the baseline trial was observed in the younger (*p* = 0.001) but not in older adults. The mean change in foot clearance in the test trial was 6.4% and 0.7% for the younger and older adults, respectively.

Scatterplots for foot clearance of the trailing limb showed the same trend as the leading limb, although the plots were relatively scattered. Contrary to those of the leading limb, the two-factor mixed ANCOVA for log-transformed foot clearance found no significant main effects of the obstacle condition (*p* = 0.568, *η*^2^ = 0.003), age group (*p* = 0.746, *η*^2^ = 0.001), and interaction between the two factors (*p* = 0.132, *η*^2^= 0.023).

## Discussion

We found that the nature of adaptive limb movement during stepping over an obstacle that had been covertly raised was the same for younger and older adults, and that they adjusted their foot clearance accordingly. More importantly, foot clearance when the obstacle was covertly lowered was significantly different between the two groups, and younger adults could adjust their leading limb movement to a lowered obstacle and lower foot clearance, while older adults did not, and their foot clearance was unchanged. These results suggested that (i) we could adjust limb movement based on subtle environmental changes irrespective of age in the case of changes that threatened safe mobility (i.e., tripping and stumbling on an obstacle), and (ii) there were age-related changes in adaptive leading limb movement in relation to covertly lowered obstacles (i.e., low-risk situation). Although explicit perception and action during obstacle avoidance are generally correlated (the higher we perceive the height of the obstacle, the higher our foot clearance)^17,18^, our results suggest that unconscious perception regarding information about an obstacle is also involved in controlling our stepping-over performance.

Contrary to our hypothesis, adaptive limb movement was observed in both groups when height of the obstacles was raised covertly; however, foot clearance in the descending condition did not change among older adults, while it did in younger ones. A possible reason for the age-related change in adaptive limb movement observed in only the descending condition is the difference in the magnitude of behavioral risk. If anticipatory adjustments are not made by modulating foot clearance in the ascending condition, the likelihood of tripping on the obstacle increases. This speculation is supported by a previous finding that the foot clearance was affected by the higher height of obstacles when we stepped over different adjacent obstacle heights using the right (leading) and left (trailing) feet^19^, suggesting that the step-over action is predominantly controlled by the safety-first principle. In contrast, we are not at risk of tripping or falling in the descending condition even if we do not make anticipatory adjustments according to the change in the obstacle height (i.e., lowered foot clearance). Our interpretation suggests that we might be sensitive to environmental changes that threaten safety to avoid potential accidents. Considering that risk perception is shaped by several largely unconscious emotional processes^20,21^, unconsciously perceived threats caused by the subtle environmental change might have driven conservative motor reactions. Further research is required to investigate this hypothesis.

Our adaptive locomotor system monitors the environmental features and predicts the appropriate adjustments required to negotiate any hazards and maintain movement^2,22,23^. During avoidance movements, it is considered that the adjustments are calibrated based on previous experiences and are generally adopted to prevent tripping or stumbling on an obstacle while minimizing energy costs^24–26^. In this study, participants adapted and raised their foot clearance during the ascending condition even though most of them did not necessarily have to do so because the clearance was sufficient during the baseline trial to step over the obstacle in the test condition (Figs. 1 and 2). One possible interpretation is that, as mentioned, adaptive movement is conservatively controlled with safety first in high-risk situations, irrespective of energy efficiency. In contrast, in low-risk situations (i.e., the descending condition), adaptive movement might be performed according to energy efficiency as well as safety. However, among older adults who experience a decline in the ability to control fine limb movement due to aging, it is better for safe locomotion to control limb movement conservatively (i.e., keep their limb movement the same as in the baseline trial) than to adjust it for energy efficiency, because the alteration may cause tripping such as by lowering their foot too much. This is justified given that some studies have demonstrated that young adults who can process defense reactions quickly showed lower foot clearance than older adults who are thought to lack the ability of avoidance behavior^8–10^. In this study, the age-related difference in foot clearance in the descending condition might have resulted from these unconscious movement strategies.

The present study showed that participants could adjust limb movement without awareness of changes in obstacle height during the step-over action, suggesting that visually guided locomotion in some situations was achieved independently of awareness. Although these results have important implications for motor control, caution should be exercised when interpreting them, as a lack of awareness does not necessarily indicate a lack of cognitive and perceptual processing. In traffic psychology, there have been cases where participants adapted their behavior to stimuli such as speed limit and route instruction signs while unable to report detection of these stimuli^27,28^. A previously mentioned recent study showed that none of the participants had bumped into the signboard, even though over half of participants had been unaware of it^15^, suggesting that the signboard’s presence has been sufficiently processed to enable the participants to successfully negotiate the obstacle. Findings from this category of research raise the possibility that objects may guide a person’s behavior without conscious awareness, and the subsequent behavior is performed without much cognitive deliberation^29,30^.

This idea is also supported by a finding related to neurological patients with selective damage to visual perception. The study demonstrated that a patient who developed profound visual agnosia could step over obstacles during locomotion as efficiently as controls, even though she could not correctly recognize the objects^31^. This result indicated that although she was unable to correctly perceive the size, shape, and orientation of the objects, her visuomotor outputs remained quite sensitive to the same object features (i.e., a damaged ventral “perception” stream but a functionally intact dorsal “action” stream). This finding suggests that visual information regarding external objects, required for the production of motor behavior, is independent of the visual information required for simple recognition^2^.

In contrast to the leading foot, no significant changes were observed in trailing foot clearance during the descending condition for both groups. Considering that the degree of change for the leading foot in the descending condition among younger adults was slightly smaller than that in the ascending condition, the result can be interpreted to indicate that subtle environmental changes in low-risk situations have less effect on the alteration of limb movement compared with high-risk situations, and this tendency is more pronounced in the trailing foo. Because stepping over an obstacle with the trailing limb is a movement that is not visible, and thus is guided by working memory of the obstacle height^32,33^, the trailing limb has been thought to be controlled differently from the leading limb when stepping over an obstacle. Although such differences in limb control during obstacle negotiation may also appear in the lack of significant alteration in trailing limb clearance, further studies will be needed to elucidate likely reasons for these discrepancies for the leading and trailing feet.

As one of the main reasons for falls is tripping while stepping over an obstacle^34^, the ability to avoid obstacles in the environment that changes from moment to moment is an important motor skill that allows safe locomotion, particularly in older adults^34,35^. Our findings may help to clarify how we adapt and regulate stepping-over action in a subtly changing environment and how this ability changes with age. However, some limitations of this study need to be outlined. Our sample comprised relatively high functioning older adults (mean gait speed > 1.0 m/s and mean MMSE > 28); therefore, selection bias in our population can limit the generalizability of the present results. Also, participants stepped over an obstacle in four steps in the present experiment. Since this relatively short approaching distance limits the situation and is different compared to approaching from a longer distance (i.e., a distance of more than four steps), caution should be exercised in interpreting our results. To overcome these limitations, further research is needed to confirm whether age-related changes in adaptive limb movement observed in the present study contribute to an increased risk (i.e., tripping and stumbling) during obstacle avoidance behavior among older adults.

We conclude that we can adjust our movement based on the subtle environmental changes irrespective of age during changes that threaten safe behavior (i.e., tripping and stumbling on an obstacle). However, the adaptive limb movement in low-risk situations (i.e., covertly lower obstacles in the present study) may be affected by aging. Our findings contribute to improved understanding of the nature of the adaptive limb movement during obstacle avoidance in response to subtle environmental changes, and how this adaptive locomotion changes with age.

## Material and methods

### Participants

The present study was modeled after a previous study^36^. Potential participants aged 65 years and older were drawn from a database of community-dwelling older adults available at the Tokyo Metropolitan Institute of Gerontology (TMIG) and contacted by mail. To be included, older adults had to be able to walk independently for 400 m without any mobility aids. We assessed the TMIG Index of Competence, a questionnaire that consisted of three multidimensional subscales: IADL, intellectual activity, and social function^37^, and confirmed that participants also had to be fully functional in IADL. Exclusion criteria included history of neurological disorders affecting motor behavior (e.g., musculoskeletal disease), history of hospitalization due to an acute medical condition within three months before the study, insufficient vision—detected by self-reported visual impairment—to identify the experimental device, significant hearing loss, and mental disorders (e.g., depression symptoms) or significant cognitive impairment (MMSE score < 24). As a result, 104 older adults (mean age and SD: 78.2 ± 5.6 years and 81.9% female) participated in the study. We also recruited young participants, free from any impediments to normal locomotion as verified by self-report, from a university population by newsletter; altogether, 104 young adults (mean age and SD, 27.5 ± 7.5, 52.9% female) participated in this study.

The study was conducted in accordance with the principles of the Declaration of Helsinki and all its amendments. All participants signed an informed consent form prior to the start of the experiment. The study protocol was approved by the local institutional review board of the TMIG Ethics Board.

### Experimental setup and apparatus

The experiment was conducted in a sound-isolated flat room illuminated with homogeneous white light. A standard white obstacle was made with expanded polystyrene, which measured 150 mm × 600 mm × 10 mm (height × width × depth), with L-brackets attached to the bottom that held the obstacle upright. The color of the floor was dark gray to highlight the obstacles. We used two test obstacles, which included heights of 165 mm or 135 mm (a 10% ascent or descent from the height of the standard obstacle), to examine the ability of adaptive limb movement to change according to the obstacle height during avoidance. The obstacle was first placed at a distance of 140 cm from the participant because of the distance needed to step over it in four steps (see *Procedure*).

Participants were outfitted with three infrared reflective markers (9.5 mm diameter) on each flat walking shoe provided by the experimenter (i.e., all the same type of shoes) to estimate the toe and heel position; they were placed on the first and fifth toes and the center of the heel on each foot. In addition, two markers were placed on the lateral side of the upper front edge of the obstacle. A three-dimensional motion capture system (OptiTrack V120: Trio, NaturalPoint, Inc.) was located diagonally on the right side in front of the participant, 160 cm high on a tripod with a sampling frequency of 120 Hz. Data were corrected using Motive software (NaturalPoint, Inc.) and analyzed using MATLAB R2017b software (MathWorks Inc., MA, USA). Time series data for each marker were smoothed by a second-order Butterworth low-pass filter with a 10 Hz cutoff frequency. Prior to the experiment, participants were allowed to become familiar with the shoes and the environment.

### Procedure

**Figure 3:**
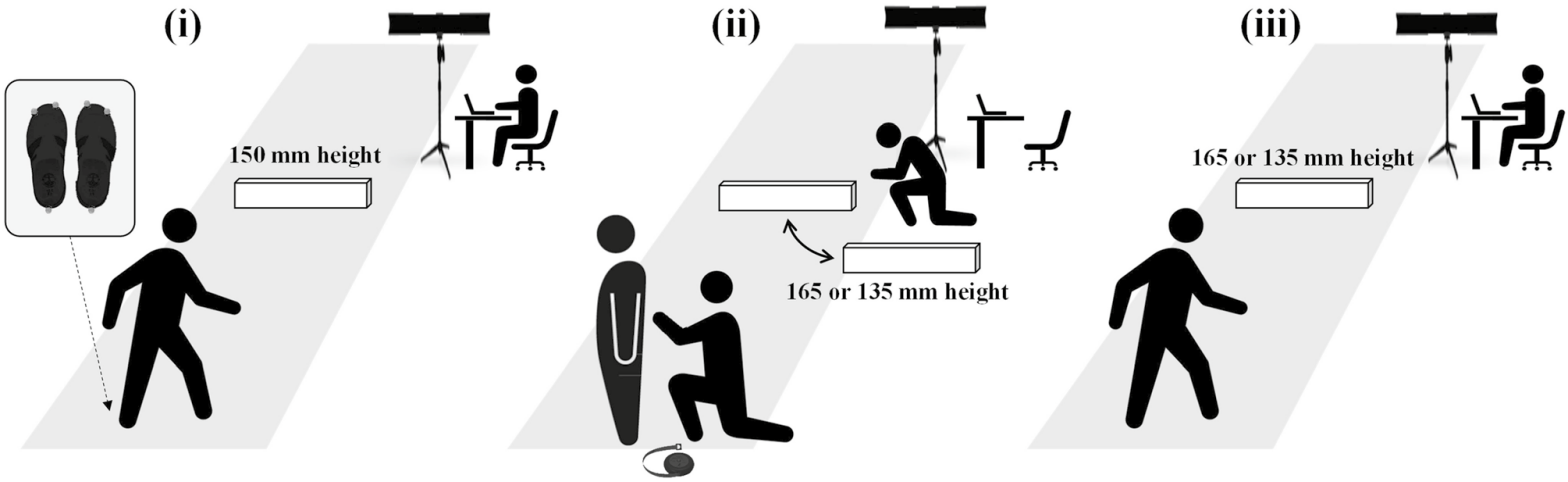
Schematic illustration of the obstacle avoidance task. (**i**) Participants walked down the pathway at a self-selected stride and stepped over the obstacle four consecutive times (i.e., baseline trial); (**ii**) an experimenter then covertly switched the obstacle to the test obstacle (i.e., 135 mm or 165 mm height obstacle) according to the participants’ allocation when another experimenter measured the length of lower limb of the participants where they could not see the obstacle; (**iii**) finally, participants were asked to step over the obstacle again as a test trial.

Participants first stepped over the standard obstacle (150 mm height) as a baseline trial. Then, the heights of the obstacles were covertly changed as a test trial and the participants were asked to step over them again (Fig. 3).

Before data collection, participants were assigned randomly to one of two groups: those who would step over only an obstacle that was covertly raised (i.e., the ascending condition: *n* = 52 in each age group) or who would step over only an object that had been covertly lowered (i.e., the descending condition: *n* = 52 in each age group) for the test trial. They were instructed to walk down the pathway, starting with their left foot at a self-determined strides of three steps; step over the obstacle with the right foot as the fourth step (i.e., left foot was the trailing foot); and then keep walking until they reached the end of the line. This four-step protocol for crossing obstacles is based on the fact that visual information during this approach is used in a feed-forward manner and obstacle avoidance during walking is planned two steps before crossing the obstacle^2,13^. For each participant, several practice trials (i.e., two to four trials) were conducted before the experiment began until their stepping-over action was stabilized, and the starting position was adjusted so that the leading foot was the right foot.

For the baseline trial, participants were asked to step over the standard obstacle (150 mm height) four times consecutively. They were then asked to face backward so they were unable to see the obstacle, and the experimenter measured the length of the lower limb (i.e., distance from the greater trochanter to the ground through the lateral malleolus). During this period, another experimenter covertly switched out the obstacle for one of the test obstacles (i.e., 135 mm or 165 mm height obstacle) based on to the participants’ assignment (i.e., ascending or descending conditions). After the length of the lower limb was measured (and the obstacle had been switched), participants were asked to step over the obstacle again as a test trial. In this case (i.e., when participants were asked to perform the test trial), we told the participants that “there was incomplete data in the previous trial and you should step over the obstacle once more” because we did not want them to notice that we had changed the obstacle. During baseline and test trials, participants did not receive any feedback for the results and were not informed that the experiment was divided into two different trials. After the test trial, we confirmed whether the participants had noticed changes in the obstacle height in the fifth trial (i.e., test trial) and excluded those who noticed the changes in accordance with our study purpose.

In this study, we measured (i) leading foot clearance, the vertical distance between the toe tips detected by the reflective markers attached to the first and fifth toes of the leading limb (first limb to pass over the obstacle) and the upper edge of the obstacle as each respective marker passed over the obstacle; and (ii) trailing foot clearance, the vertical distance between the toe tips of the trailing limb (the second limb to pass over the obstacle) and the top of the obstacle as the foot passed over the it.

### Other variables

To show physical and cognitive characteristics of older samples, we assessed gait speed and global cognitive function. For gait speed, a trained tester asked participants to walk once along an 11-m straight walkway on a flat surface at their usual strides. We used a stopwatch to measure the elapsed time for walking 5 m, the center of the 11-m pathway (i.e., a steady state of walking), and calculated the speed. The first and last 3 m were considered as the acceleration and deceleration phases and were not in the speed calculation. Global cognitive functioning was assessed using the MMSE, which has a maximum score of 30 points, with higher scores indicating better overall cognitive functioning.

### Statistical analysis

Descriptive statistics of the differences in participants between ascending and descending conditions in each age group were examined using χ^2^ tests for the categorical variables and two-sample t-tests for continuous variables. For foot clearance in the baseline trial, we used the mean foot clearance, which included the first to fourth trials. A two-factor mixed analysis of variance (ANOVA) was conducted on foot clearance with two independent factors, including the trial (baseline and test trials, i.e., a within-subjects factor) and age (younger and older adults, i.e., a between-subject factor), to confirm age-related differences in adaptive limb movement when stepping over after a subtle change was made in obstacle height. This was performed separately for each obstacle condition (i.e., the ascending and descending conditions) and variable (i.e., leading and trailing limbs). Each ANOVA was adjusted for gender and length of the lower limb (e.g., ANCOVA) because they are considered as possible covariates affecting foot clearance. Furthermore, because we did not confirm parametric distributions in trailing foot clearance in each obstacle condition and age group using the Shapiro–Wilk test (*p* < 0.05), log transformations were performed on them to normalize the distributions (i.e., ANCOVAs on the trailing foot were performed on log-transformed values, whereas ANCOVAs on the leading foot were performed on actual values). If significant interaction between two factors was observed, post-hoc tests with Bonferroni correction were adopted. Statistical analyses were performed using SPSS version 23 (IBM Inc., Chicago, IL, USA). Significance level was set at *p* < 0.05.

## Data Availability

The datasets analyzed during this study are available from the corresponding author upon reasonable request and after approval by institutional authorities.
